# The Interactions Between Circadian Rhythm, Gut Microbiota, and Anxiety: From Mechanisms to Intervention Strategies

**DOI:** 10.3390/nu18132209

**Published:** 2026-07-07

**Authors:** Yijin Wu, Jiaqi Wang, Lumei Kang, Xiaojuan Wan

**Affiliations:** 1 Department of Histology and Embryology, School of Basic Medical Sciences, Jiangxi Medical College, Nanchang University, Nanchang 330006, China; 2 Queen Mary School, Jiangxi Medical College, Nanchang University, Nanchang 330006, China

**Keywords:** circadian rhythm, gut microbiota, anxiety, neuroendocrine, immune regulation, chrononutrition

## Abstract

The circadian rhythm is an internal timing system formed by the body’s adaptation to the Earth’s rotation, which helps maintain homeostasis by regulating physiological, metabolic, and behavioral activities. The gut microbiota (GM), the largest microbial ecosystem in the human body, exhibits a bidirectional regulatory relationship with the host circadian clock. Emerging evidence indicates that circadian rhythm disruption (CRD) is linked to disturbances in the diurnal oscillations and compositional balance of the GM, accompanied by reduced short-chain fatty acid levels, increased lipopolysaccharide leakage, and altered tryptophan metabolism. These microbial abnormalities may be involved in anxiety-like behaviors through three major pathways: neuroendocrine (hyperactivation of the HPA axis), immune (microglia-mediated neuroinflammation), and neurotransmitter (imbalance of the serotonergic and dopaminergic systems). Conversely, microbial metabolites such as butyrate and secondary bile acids may reciprocally regulate peripheral clock gene expression, forming a complex “circadian rhythm–GM–anxiety” interaction network. This review summarizes the molecular basis of circadian–GM interactions, potential GM-mediated mechanisms linking CRD with anxiety, and emerging intervention strategies including chrononutrition (time-restricted feeding, sequential nutrient intake), microbiota-targeted therapies (probiotics/prebiotics, fecal microbiota transplantation), and light therapy and melatonin supplementation. Future directions should focus on cell-specific mechanisms using single-cell and spatial transcriptomics, developing personalized interventions that integrate chronotype and microbiome profiling, and conducting large-scale randomized controlled trials to facilitate clinical translation. This review provides a framework for understanding the integrative role of circadian biology and gut microbiota in anxiety and may help develop precision intervention paradigms.

## 1. Introduction

The circadian rhythm refers to the approximately 24 h fluctuations in life activities observed in most organisms, which are synchronized with the solar day [1]. The circadian rhythm system is essential for maintaining the coordination between internal physiology, behavior, and external environmental cues (such as sunlight). When this synchronization is disrupted, circadian rhythm disorders (CRDs) occur [2].

The modern lifestyle of busy individuals is often characterized by exposure to artificial light at night from lamps, computer screens, mobile phone screens, shift work, and reduced daytime sunlight exposure. These factors may contribute to CRDs and have also been associated with mental health problems [3]. A cohort study encompassing 175,543 employed or self-employed individuals revealed that shift work was associated with mental health outcomes, including anxiety, and the risk of anxiety increased with greater shift-work frequency [4]. A meta-analysis of longitudinal studies further showed that shift work was associated with an elevated overall risk of adverse mental health outcomes, particularly depressive symptoms [5]. 

The gut microbiota (GM) represents the largest microbial reservoir in the human body, comprising approximately 100 trillion bacteria. Most of these bacteria belong to the Bacteroidetes and Firmicutes phyla [6]. The GM coexists with the host in a symbiotic manner and includes at least 1000 species, regarded as the largest and most direct external environment of the human body [7]. The GM plays crucial roles in metabolism and nutrition, pathogen resistance, immune regulation, brain development, and neural development [8]. Numerous studies have demonstrated that the composition and function of the GM in patients with CRDs or mental disorders differ from those of normal individuals [9,10].

A complex bidirectional regulatory pathway exists between the gut and the brain, known as the “gut–brain axis” [11]. Given the important role of the GM, this axis is also referred to as the “microbiota-brain–gut axis” [12]. This axis primarily involves the autonomic nervous system, enteric nervous system, immune system, and endocrine system (including the hypothalamic–pituitary–adrenal axis), and facilitates bidirectional communication via signaling molecules such as neurotransmitters, cytokines, hormones, and microbial metabolites [13].

For this review, human observational studies are taken as evidence of association, not evidence of causation. Animal experiments, microbial-transfer studies, and mechanistic studies support possible biological explanations, but are not regarded as direct clinical evidence for human anxiety disorders. Hence, the pathways discussed below are described as potential mechanisms unless supported by direct clinical or interventional evidence.

This article aims to explore the relationship and underlying mechanisms among circadian rhythm, GM, and anxiety, and to expand upon relevant research findings, thereby providing more comprehensive and in-depth information on this topic.

### 1.1. Biological Basis and Regulatory Network of Circadian Rhythms

#### 1.1.1. Molecular Mechanism of Circadian Rhythms

**Figure 1:**
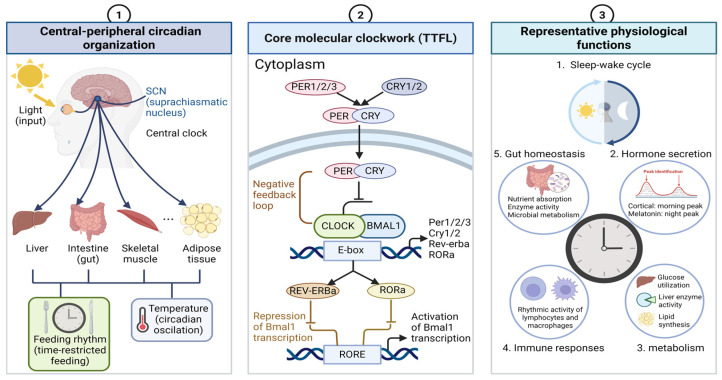
Central–peripheral organization, core molecular clockwork, and representative physiological functions of circadian rhythms.

The circadian rhythm is driven by a group of highly conserved clock genes through a transcription-translation feedback loop (TTFL) [14]. Circadian Locomotor Output Cycles Kaput (CLOCK) and Brain and Muscle ARNT-Like 1 (BMAL1) proteins form a heterodimer. The CLOCK/BMAL1 complex binds to E-box enhancer elements in the promoters of target genes, including Period (Per1, Per2, Per3) and Cryptochrome (Cry1, Cry2) genes, thereby driving their transcription. After entering the nucleus, the PER-CRY complex directly represses the transcriptional activity of the CLOCK/BMAL1 complex, repressing its own transcription and forming a negative feedback loop. Additionally, Rev-erbα and RORα are also target genes of the CLOCK/BMAL1 complex via E-boxes. The REV-ERBα and RORα proteins compete for binding to RORE elements in the BMAL1 promoter (Figure 1). This loop provides stability and robustness to the core clock, generating antiphase oscillations of Bmal1 mRNA [15]. The central clock is located in the suprachiasmatic nucleus (SCN) of the hypothalamus and is synchronized by light signals [16]. Peripheral clocks (e.g., in the liver and intestine) are regulated by non-light signals such as feeding and temperature [17]. For example, a study in mice showed that altering feeding time significantly affected the expression rhythm of clock genes in the liver, demonstrating that peripheral clocks can be independently regulated by non-light factors [18].

#### 1.1.2. Physiological Function of the Circadian Rhythm

The circadian rhythm coordinates sleep–wake cycles, hormone secretion (e.g., cortisol, melatonin), metabolic activities (e.g., glucose utilization, lipid synthesis), and immune responses [19]. For instance, cortisol peaks in the morning to promote awakening, whereas melatonin is secreted at night to induce sleep [20,21]. Peripheral clocks (e.g., in the gut) maintain local homeostasis by regulating enzyme activity, nutrient absorption, and microbial metabolism [22]. A study in human volunteers found that the activity of liver enzymes involved in glucose metabolism exhibited a clear circadian rhythm, with higher activity during the day and lower activity at night, which aligns with the body’s energy demand pattern [23]. In addition, the immune system also follows a circadian rhythm. The number and activity of immune cells, such as lymphocytes and macrophages, fluctuate throughout the day, helping the body respond more effectively to pathogens at different times [24].

#### 1.1.3. Clinical and Epidemiological Evidence Linking Rhythm Disorders and Anxiety

Accumulating clinical and epidemiological evidence supports an association between circadian disruption and anxiety-related outcomes. In a large-scale cohort with a median follow-up of 9.06 years, overall shift work exposure was associated with an increased risk of anxiety (HR 1.16, 95% CI 1.04–1.28). However, among shift workers, no significant difference in anxiety risk was observed between night-shift and non-night-shift workers, suggesting heterogeneity across different shift-work exposure patterns [25]. Sleep irregularity has also been identified as an independent risk factor, with each 1-h increase in variability of sleep duration associated with higher odds of anxiety disorders (OR 1.55, 95% CI 1.35–1.78) [25]. Consistently, meta-analytic evidence, primarily derived from youth populations, suggests that evening chronotype is modestly associated with increased anxiety symptoms (pooled r = 0.13, 95% CI 0.09–0.18), although with substantial heterogeneity (*I*^2^ ≈ 88%) and limited longitudinal support. Whether this association extends to adult populations remains less clear [26]. Experimental studies further support biological plausibility, as disruption of core circadian clock genes alters anxiety-like behaviors in animal models. For example, Cry1 knockout mice exhibit increased anxiety-like behavior, whereas Npas2 knockout mice show reduced anxiety-like responses in paradigms such as the elevated plus maze, light–dark box, and open field tests, indicating a role of circadian transcriptional regulation in emotional behavior [27]. Notably, these findings are not entirely consistent across models, likely reflecting differences in specific clock genes, brain regions, and experimental conditions.

However, these associations may also be influenced by confounding factors, including sleep-related factors, stress exposure, and metabolic alterations. Circadian disruption and sleep disturbance are closely related but should not be treated as equivalent. Disruption of the circadian rhythm mainly involves the mismatch between the endogenous circadian rhythm and external or behavioral cycles, while sleep is related to sleep duration, sleep stages, and sleep patterns [27]. In daily life, these factors often co-occur, making it difficult to determine whether changes in GM and anxiety-related outcomes are driven by CRD itself, changes in sleeping factors, or both. In this review, most reported findings are interpreted as CRD-associated. The findings can only be defined as CRD-specific effects when the original studies directly assess or statistically control for variables related to sleep and the phase of the circadian rhythm.

## 2. Methods

This review is a narrative review because the topic covers several diverse areas, including circadian biology, gut microbiota research, anxiety-related clinical findings, experimental animal models, microbiota-transfer studies, and intervention-related evidence. The aim was to integrate potential mechanisms and clinical evidence.

Relevant literature was searched between March and June 2026 using PubMed, Web of Science, and Google Scholar. Search terms included “circadian rhythm”, “circadian rhythm disruption”, “shift work”, “sleep disturbance”, “gut microbiota”, “gut microbiome”, “anxiety”, “gut–brain axis”, “microbiota–gut–brain axis”, “HPA axis”, “neuroinflammation”, “microglia”, “tryptophan metabolism”, “serotonin”, “dopamine”, “short-chain fatty acids”, “time-restricted feeding”, “probiotics”, “prebiotics”, “fecal microbiota transplantation”, “light therapy”, and “melatonin”. Different combinations of these terms were applied with Boolean operators (AND and OR). No language restriction was applied during the search.

We included studies that addressed any of our themes: circadian regulation, microbial rhythmicity, gut–brain signaling, or anxiety outcomes. Studies were selected based on their relevance to the circadian rhythm–gut microbiota–anxiety axis. Human studies were considered when available, and animal, microbiota-transfer, and mechanistic studies were included to support the discussion of potential pathways. Relevant reviews and meta-analyses were also consulted to provide background and summarize existing evidence.

Studies were excluded if they were clearly outside the scope of circadian rhythm, gut microbiota, or anxiety-related outcomes. Articles focusing exclusively on depression were not retained. Duplicate records, conference abstracts, and non-peer-reviewed reports were also excluded. As this article was not designed as a systematic review, formal PRISMA reporting, quantitative synthesis, and risk-of-bias scoring were not performed. Instead, the search process, eligibility considerations, and evidence limitations are described here to improve transparency.

## 3. Circadian Rhythm of the Gut Microbiota (GM) and Its Regulatory Mechanisms

### 3.1. Characteristics of Day-Night Fluctuations of GM

The composition and functions of the gut microbiota (GM) show 24 h periodic changes. Approximately 10–15% of microbial groups (e.g., Bacteroidetes, Firmicutes) show diurnal differences in abundance, and the activities of functional genes (such as those involved in flagellum assembly and glycosaminoglycan degradation) fluctuate over time [28]. For example, in Period2 gene knockout mice, light–dark cycle changes were associated with altered gut microbiota composition and circulating SCFA levels [29].

### 3.2. Host Factors Regulating the Circadian Rhythm of GM

#### 3.2.1. Feeding Rhythm

In Per1/2 knockout mice, time-restricted feeding (TRF) may help correct microbiota imbalance related to circadian rhythm disruptions (CRDs) [30]. Chaix and colleagues discovered that TRF is effective in preventing obesity and metabolic syndrome in mice that are genetically engineered to lack a functional circadian clock system [31]. In a mouse model of obesity induced by a high-fat diet, TRF significantly improved insulin resistance, partially mediated by the interplay between restructured GM and bile acid metabolism [32].

#### 3.2.2. Light Cycle

GM oscillation disappears in mice raised in continuous darkness, suggesting that the light cycle can influence microbial rhythmicity in experimental settings [33]. A study compared the GM composition of mice kept in a regular light–dark cycle with that of those kept in continuous darkness. The results showed that the mice in continuous darkness exhibited a significant reduction in GM diversity and a change in the relative abundance of some key bacterial groups, indicating the important role of the light cycle in regulating the GM circadian rhythm [34].

#### 3.2.3. Host Clock Genes

In homozygous Bmal1 deletion mice produced by heterozygous breeding, loss of this core clock gene was associated with changes in gut microbiota composition and a disruption of diurnal microbial oscillations, suggesting that host clock genes are involved in regulating microbial rhythmicity in mice [35].

### 3.3. Reverse Regulation of the Host Circadian Rhythm by the Gut Microbiota (GM)

The GM may feed back on host circadian regulation through microbial metabolites, such as SCFAs and secondary bile acids. In mice, oral administration of an SCFA/lactate mixture was reported to shift PER2::LUC rhythms in peripheral tissues and to alter the phase of several clock gene expression rhythms [36]. A study in mice showed that the administration of secondary bile acids could alter the expression rhythm of genes associated with energy metabolism in the liver and adipose tissue, and this effect was related to the activation of the TGR5 receptor and the regulation of GLP-1 secretion [37].

Most of the evidence provided in this section is from animal or experimental research and generally supports the biological explanations of circadian–microbiota connections. Additional human studies are necessary to determine whether these processes are preserved in diverse physiological and clinical settings. Representative human, animal, and mechanistic studies related to gut microbiota and anxiety-related mechanisms are summarized in Table 1.

## 4. Potential Mechanisms Linking CRD and Anxiety

**Figure 2:**
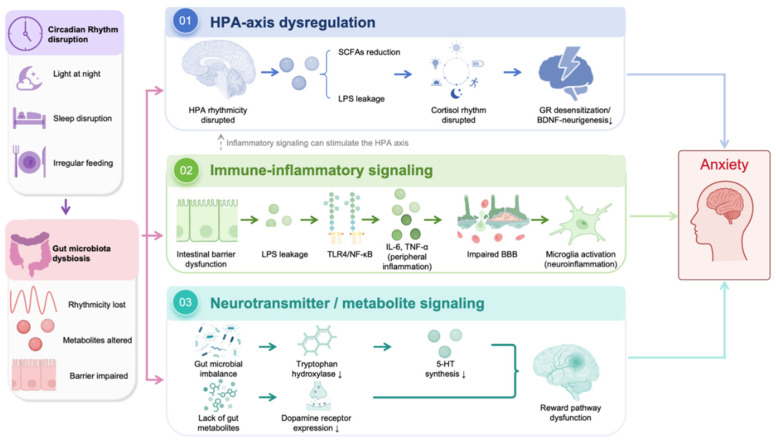
Three potential gut–brain pathways linking circadian rhythm disruption, gut microbiota dysbiosis, and anxiety. Different colors are used only to distinguish the three pathways, and arrows indicate possible directions of interaction without implying confirmed causal relationships.

CRD may alter gut microbial rhythmicity, microbial metabolite production, and intestinal barrier integrity. These changes are proposed to contribute to anxiety through three interacting gut–brain pathways. First, CRD disrupts SCN-mediated HPA-axis rhythmicity, while gut microbiota dysbiosis is associated with reduced short-chain fatty acids (SCFAs) and increased lipopolysaccharide (LPS) leakage. These factors may contribute to cortisol rhythm disruption, glucocorticoid receptor desensitization, impaired BDNF-related neurogenesis, and increased risk of anxiety. Second, intestinal barrier dysfunction may facilitate LPS leakage, followed by TLR4/NF-κB activation, increased IL-6 and TNF-α production, peripheral inflammation, impaired blood–brain barrier integrity, and microglial activation. Third, gut microbiota imbalance may reduce tryptophan hydroxylase activity and 5-HT synthesis, while lack of gut microbiota-derived metabolites may reduce dopamine receptor expression and impair reward-related signaling. The three pathways are shown separately for clarity but are likely to interact in vivo. CRD is difficult to separate from sleep disturbance, stress exposure, diet, and medication use. These factors may also affect GM changes and anxiety-related symptoms independently. The three potential mechanisms are summarized in Figure 2 and should be studied further.

### 4.1. Definition and Causes of Circadian Rhythm Disruption (CRD)

CRD refers to the desynchronization of central and peripheral clocks, which can be caused by genetic mutations, shift work, travel across time zones, or sleep deprivation. CRD has been linked to metabolic disorders, immune abnormalities, and neuropsychiatric symptoms [47].

It has been reported that mice carrying the ClockΔ19 mutation exhibit altered anxiety-related behaviors compared to control mice [48]. This animal evidence provides biological evidence for a link between CRD and anxiety behaviors, but it should not be interpreted as direct evidence for human anxiety disorders. In addition, in humans, long-term shift work has been associated with an increased risk of anxiety disorders [49]. A multi-center cross-sectional study, which included 11,061 nurses from 20 different hospitals, revealed that among nurses working shifts, 62.08% showed signs of anxiety. Furthermore, these prevalence rates were found to be influenced by the level of fatigue experienced during shift work [50]. 

### 4.2. Potential Pathways Linking CRD, GM Dysregulation, and Anxiety

#### 4.2.1. Neuroendocrine Pathway: Overactivation of the Hypothalamic-Pituitary-Adrenal (HPA) Axis

CRD may interfere with SCN-mediated regulation of the HPA axis, potentially disrupting the secretion rhythm of cortisol (CORT) [51]. High levels of cortisol may induce anxiety through the following mechanisms:

Glucocorticoid receptor (GR) desensitization: Long-term exposure to CORT has been reported to reduce the sensitivity of GR, weaken the negative feedback regulatory mechanism, and lead to continuous activation of the HPA axis [52]. A study in mice with forebrain-specific disruption of glucocorticoid receptors showed alterations in anxiety-related behavioral patterns and stress-induced HPA-axis activation. These findings suggest that impaired GR signaling in forebrain regions may weaken central feedback control of the HPA axis, thereby contributing to abnormal stress responses and anxiety-like behaviors [53].

Inhibition of hippocampal neurogenesis: In healthy young men, stronger cortisol responses to acute psychosocial stress were associated with a faster post-stress decline in brain-derived neurotrophic factor (BDNF). The authors proposed that chronically elevated cortisol levels could contribute to reduced neurogenesis under chronic stress conditions [54]. A clinical study reported that lower BDNF levels were associated with anxiety symptom severity, suggesting that BDNF-related pathways may be involved in anxiety-related outcomes [55].

GM imbalance may exacerbate HPA axis dysregulation through the following pathways:

Reduction in SCFAs: The deficiency of short-chain fatty acids (SCFAs) may weaken the inhibition of the HPA axis and promote the secretion of CORT [56]. A study on mice with antibiotic-induced GM dysbiosis found that the levels of butyrate in the feces were significantly reduced, while at the same time the secretion of CORT was increased, supporting a potential role of butyrate on the HPA axis [57].

Lipopolysaccharide (LPS) leakage: When LPS enters the bloodstream, it can activate the TLR4/NF-κB pathway, thereby elevating the release of pro-inflammatory cytokines, which may further engage the HPA axis. In diabetic animal models, gut microbiota disturbance was also linked to endotoxin/TLR4-related inflammatory activation, suggesting that this pathway may link GM dysbiosis with HPA-axis disturbance [58]. A study on patients with functional dyspepsia found that an increase in intestinal permeability leads to the entry of LPS into the bloodstream, which is associated with overactivation of the HPA axis and an increased risk of anxiety-related symptoms [59].

#### 4.2.2. Immune Pathway: Neuroinflammation and Microglial Activation

Inflammation is involved in both physiological and pathological processes, and the inflammatory response is essential for maintaining homeostasis and restoring tissue function [60]. The biological clock regulates the secretion of inflammatory cytokines [61]. Under normal conditions, cytokines such as interleukin-1β (IL-1β), IL-6, and TNF-α show circadian variation [62]. Sleep disturbance and CRD are closely related but not identical, and their effects on inflammatory signaling may overlap. Previous evidence suggested reciprocal interactions among neuroinflammation, sleep regulation, and circadian rhythms [63]. Therefore, the inflammatory changes discussed below should not be attributed to CRD alone, as sleep loss or poor sleep quality may also influence immune activity, GM composition, and anxiety-related symptoms.

Recent studies in mice suggest that circadian disruption may also affect intestinal barrier function and endotoxemia, while altering gut microbial composition and intestinal homeostasis [63]. Gut microbiota-derived signals are closely involved in this process. Butyrate has anti-inflammatory effects and can suppress NF-κB activation by inhibiting IκBα degradation. Receptor-mediated pathways related to SCFAs, including GPR109A, are also involved in mucosal immune regulation. LPS may enter the circulation more easily when intestinal barrier integrity is impaired. It binds to CD14 and MD2 and activates TLR4, followed by MyD88- and TRIF-dependent signaling, which increases NF-κB activity and promotes the production of pro-inflammatory cytokines [64]. Animal studies have also shown that inflammatory responses to LPS vary across the day, indicating that immune activity is regulated by both central and peripheral clocks [65].

This regulation is also present in the central nervous system. Experimental studies suggest that microglia show time-of-day variation in inflammatory state, and the clock gene Bmal1 is involved in regulating microglial immune function and phagocytic activity [66]. Peripheral inflammatory signals can reach the brain through several pathways, including vagal afferents, active cytokine transport across the blood–brain barrier (BBB), and diffusion through circumventricular organs [67]. Systemic inflammation may also impair BBB integrity and facilitate the entry of circulating cytokines and inflammatory mediators into the central nervous system. These changes may contribute to microglial activation and sensitization. Activated microglia can produce pro-inflammatory mediators, including TNF-α, IL-1β, nitric oxide (NO), and reactive oxygen species (ROS), which may further amplify neuroinflammatory signaling [68].

Inflammatory factors are not only associated with CRD but also with mental health disorders [69]. TNF-α and IL-6 can affect neurotransmitter metabolism, neural plasticity, and neuroendocrine systems during chronic inflammation, thereby contributing to anxiety-related symptoms [70]. Taken together, gut microbiota-related inflammatory signaling and microglial activation may be one of the main immune mechanisms linking CRD to anxiety-related outcomes.

#### 4.2.3. Neurotransmitter Pathways: Imbalance of Monoamine Systems

CRD has been linked to alterations in the synthesis and release of monoamine neurotransmitters (e.g., serotonin (5-HT), dopamine) through GM dysfunction:

5-HT deficiency: The GM imbalance has been reported to reduce the activity of tryptophan hydroxylase and decrease 5-HT synthesis [71]. A study in mice with alcohol dependence showed that inulin can elevate the populations of *Faecalibacterium* and *Roseburia* bacteria, boost the production of SCFAs, and modulate 5-HT metabolism. These effects were accompanied by the alleviation of anxiety-like behaviors [72]. However, whether similar effects occur in primary anxiety disorders in humans remains unclear.

Dopaminergic system inhibition: The lack of GM metabolites (such as SCFAs) has been associated with reduced expression of dopamine receptors, which may lead to dysfunction of the reward pathway and manifest as loss of pleasure and anxiety [73]. Solleiro and colleagues proposed that the heightened anxiety levels seen in rats with streptozotocin-induced diabetes are associated with the activity of dopamine D1 receptors [74].

## 5. Intervention Strategies Based on the Circadian-GM Axis

**Figure 3:**
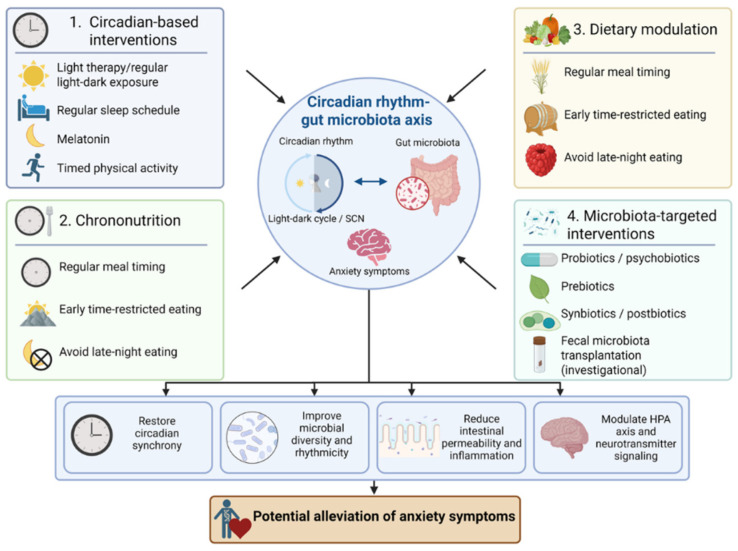
Overview of potential intervention strategies targeting the circadian rhythm–gut microbiota axis in anxiety. The figure summarizes circadian-based interventions, chrononutrition, dietary modulation, and microbiota-targeted interventions and their possible downstream effects.

As shown in Figure 3, interventions targeting the circadian rhythm–GM axis mainly include circadian-based interventions, chrononutrition, dietary modulation, and microbiota-targeted interventions. These approaches may help restore circadian synchrony, improve gut microbiota diversity and rhythmicity, reduce intestinal permeability and inflammation, and regulate HPA-axis and neurotransmitter signaling. In this section, we focus on several interventions most relevant to the circadian rhythm–GM axis, including time-restricted feeding, sequential nutrient intake, probiotics and prebiotics, fecal microbiota transplantation, light therapy, and melatonin supplementation.

### 5.1. Chrononutrition (Time Nutrition)

#### 5.1.1. Time-Restricted Feeding (TRF)

TRF is a dietary intervention that limits the eating period to 8 to 10 h each day. It usually aligns the eating window with the body’s natural circadian rhythm, such as from 10:00 AM to 6:00 PM. This method has shown favorable results in restoring the regularity of the gastric motility cycle [75]. The gastric motility cycle, like many other physiological processes in the human body, follows a circadian rhythm, and the disruption of this rhythm is associated with various health problems, including metabolic syndrome and anxiety disorders [76].

Animal experiments have provided valuable insights into the mechanisms underlying the potential benefits of TRF. In mouse models of chronic jet lag, TRF has been reported to act as an entrainment mechanism. It was reported to correct disruptions in the circadian clock and the metabolically related transcriptome within the jejunal mucosa, primarily through the regulation of BMAL1 [77]. At the same time, TRF has also been reported to promote the generation of SCFAs by the GM [78]. Moreover, in dextran sulfate sodium-induced ulcerative colitis mice, TRF restored gut microbiota composition, increased the Firmicutes/Bacteroidetes ratio, and enriched *Akkermansia*. Concurrently, it reduced the relative populations of *Citrobacter*, *Bacteroides*, and *Escherichia-Shigella.* Furthermore, in this mouse model, TRF attenuated the occurrence of associated behavioral abnormalities, a phenomenon closely linked to the coordinated regulatory changes within the gut–brain axis [79]. However, whether these effects can be translated to clinical anxiety disorders remains unclear because equivalent human randomized controlled trials are lacking.

#### 5.1.2. Sequential Nutrient Intake

The concept of sequential nutrient intake involves consuming different types of nutrients at specific times of the day to optimize their effects on the body’s circadian rhythm and mental health. Eating foods rich in tryptophan at night is a key component of this strategy. Tryptophan is an essential amino acid that serves as the foundational building block for the synthesis of 5-HT. 5-HT is a neurotransmitter critically involved in the regulation of mood, sleep patterns, and anxiety levels [80]. Foods such as milk and nuts are good sources of tryptophan. Consuming tryptophan-rich foods at night may support serotonin-related pathways and sleep regulation, but direct evidence that this strategy reduces anxiety symptoms remains limited [81]. On the other hand, consuming high-fiber foods during the day may support emotional regulation. High-fiber foods are fermented by intestinal bacteria in the large intestine, producing SCFAs. Increased SCFA production may influence emotional stability and anxiety-like behaviors, but current evidence remains largely preclinical and may not directly establish anxiety prevention in humans [82].

### 5.2. Microbiota-Targeted Interventions

#### 5.2.1. Probiotics and Prebiotics

Probiotics refer to live microorganisms that, when consumed in sufficient quantities, provide health benefits to the host [83]. In contrast, prebiotics are non-digestible food ingredients that specifically promote the growth and enhance the activity of beneficial bacteria within the gut [84]. Supplementation with probiotics or prebiotics has been studied for its potential effects on gut microbiota composition and related gastrointestinal outcomes [85]. One of the main effects is the restoration of the diversity of GM. A diverse GM is essential for maintaining gut health and overall well-being. Disruptions of the microbiota diversity have been associated with various diseases, including anxiety disorders. By introducing beneficial bacteria or providing nutrients for their growth, probiotics and prebiotics can help restore the balance and diversity of the microbiota [86]. In addition, probiotics and prebiotics may influence the overactivity of the hypothalamic–pituitary–adrenal (HPA) axis. The HPA axis is an important neuroendocrine system that regulates the body’s response to stress. Overactivity of the HPA axis is more common in individuals with anxiety disorders, potentially leading to excessive secretion of the stress hormone cortisol [87]. By modulating the HPA axis, probiotics and prebiotics can help lower stress levels and alleviate anxiety symptoms [88]. A systematic review and meta-analysis of controlled clinical trials showed that probiotics have antidepressant and anxiolytic effects [89]. Zhu and colleagues proposed that administering the psychobiotic *Lactobacillus plantarum* JYLP-326 represented an effective approach for mitigating anxiety, depression, and insomnia among college students experiencing test-related stress. The underlying mechanism of this effect may be associated with the modulation of gut microbiota composition and fecal metabolite profiles [90]. However, these effects may vary across probiotic strains and individual baseline conditions, and current evidence does not support a uniform effect across all anxiety disorders.

#### 5.2.2. Fecal Microbiota Transplantation (FMT)

FMT is a process that involves transferring the fecal microbiota from healthy donors into the gastrointestinal tract of patients [91]. Altered GM composition has been reported in anxiety-related conditions. In a mouse model receiving fecal microbiota from patients with generalized anxiety disorder, FMT induced anxiety-like behaviors and was associated with changes in endocannabinoid-related signaling [46].

In addition to restoring the rhythmicity of the gut microbiota, FMT may also influence HPA axis function and emotional symptoms. The HPA axis is closely linked to the gut microbiota, and a disorder in one may affect the other. By restoring a normal gut microbiota through FMT, it is possible to regulate the HPA axis and alleviate stress-related symptoms [92].

Animal studies suggest that FMT can affect brain-related signaling. Studies have shown that FMT can upregulate the expression of brain-derived neurotrophic factor (BDNF). BDNF is a protein that plays a crucial role in the survival, growth, and differentiation of neurons. It is also involved in neurogenesis, the process of generating new neurons in the hippocampus [93]. In a mouse study, transplantation of fecal microbiota from patients with alcoholism induced anxiety- and depression-like behaviors and decreased brain mGluR1/PKC ε levels [94]. Overall, these findings indicate that FMT can influence microbiota–brain signaling, but its effects in humans remain unclear.

### 5.3. Light Bioregulation and Melatonin

#### 5.3.1. Light Therapy

Light therapy, particularly short wavelength blue light therapy (BLT), has been studied as an approach for regulating circadian rhythm and sleep–wake timing [95]. Exposing individuals to blue light in the morning has multiple beneficial effects. First, it can inhibit the secretion of melatonin. Melatonin is a hormone secreted by the pineal gland and is responsible for modulating the sleep–wake cycle [94]. Typically, melatonin increases at night to promote sleep and decreases in the morning to help people wake up. By inhibiting the secretion of melatonin in the morning, BLT can help reset the clock of the suprachiasmatic nucleus (SCN), which is the main circadian rhythm clock in the brain [96]. Second, resetting the SCN clock can improve the sleep–wake cycle. A regular sleep–wake cycle is crucial for maintaining good mental health, and disruptions to this cycle are often associated with anxiety and other emotional disorders. In addition to improving the sleep–wake cycle, BLT can also positively affect emotional states. By improving sleep–wake timing and circadian alignment, BLT may indirectly influence emotional states, although direct evidence for reducing anxiety symptoms remains limited [97]. Furthermore, BLT combined with time-restricted feeding (TRF) may have a synergistic effect on restoring the brain’s rhythm. The circadian rhythm of the gastric mucosa is influenced by both dietary factors and light exposure. By combining these two intervention strategies, it may be possible to more effectively regulate the circadian rhythm of the gastric mucosa, thereby improving gut health and mental health [98]. However, whether a similar combined light and TRF strategy can improve gut health or anxiety-related outcomes in humans remains unclear.

#### 5.3.2. Melatonin Supplementation

Melatonin supplementation is another method for regulating the circadian rhythm and improving mental health [99,100]. By activating the MT1/MT2 receptors, melatonin has been reported to suppress the activity of the HPA axis [101]. Through this inhibition, melatonin is associated with reduced cortisol secretion and may help alleviate anxiety symptoms [102].

In addition to its effect on the HPA axis, melatonin may also regulate the gut microbiota composition [103]. Studies have shown that melatonin supplementation can increase the abundance of certain beneficial bacteria, such as *Akkermansia* [104]. In mice exposed to water-avoidance stress and sleep deprivation, melatonin treatment attenuated stress-induced dysbiosis and increased the abundance of *Lactobacillus* and *Akkermansia muciniphila* [105].

## 6. Discussion and Prospects

### 6.1. Current Limitations

Current evidence suggests a possible link among circadian rhythm disruption, gut microbiota dysbiosis, and anxiety, but this relationship remains incompletely established. Most human microbiome studies in anxiety disorders are small, observational, or cross-sectional, making it difficult to determine whether microbial alterations are a cause, consequence, or correlate of anxiety [106]. Circadian disruption also often overlaps with sleep disturbance, psychosocial stress, irregular feeding, medication exposure, and metabolic changes, which further limit causal interpretation [107].

Taxonomic findings also vary across studies, probably because of differences in cohort characteristics, diagnostic criteria, sequencing methods, diet, and medication exposure. Thus, current microbiome evidence should be interpreted cautiously, with greater attention to functional and longitudinal changes rather than isolated bacterial groups [106,108]. Although animal and microbiota-transfer studies provide mechanistic support, their translation to human anxiety disorders remains uncertain. Intervention evidence is also preliminary, and larger controlled trials are needed to determine efficacy, safety, timing, dosage, and target populations [109,110].

These limitations are relevant to the mechanisms and interventions discussed in Section 4 and Section 5. For the mechanistic pathways, current studies often cannot determine the relative contribution of CRD, sleep disruption, and stress exposure. The outcomes they fail to clarify include changes in HPA-axis activity, inflammatory signaling, neurotransmitter metabolism, or GM composition. This limits the attribution of these pathways to CRD itself. For intervention studies, treatment effects may vary with daily diet, sleep pattern, timing, and microbiota composition. Future studies should measure circadian rhythm, sleep, diet, medication use, and stress exposure simultaneously to better distinguish these confounders.

### 6.2. Future Research Directions: Deepening Mechanism Understanding

To further advance our understanding, future research should focus on elucidating the cellular and molecular mechanisms underlying the relationship between CRD, GM, and anxiety. By employing advanced technologies such as single-cell sequencing and spatial transcriptomics, it is possible to conduct highly detailed and resolution-intensive analyses of cellular and spatial changes across tissues [111]. Single-cell sequencing can analyze the gene expression patterns of individual cells, helping to identify specific cell types and signaling pathways involved in anxiety induced by CRD and GM imbalance [112]. Spatial transcriptomics, on the other hand, can map the gene expression patterns across different regions of a tissue, thereby providing spatial organization information about cellular processes related to anxiety [113]. By combining these technologies, researchers can gain a more comprehensive understanding of the complex interactions between the circadian rhythm and GM in the brain and develop more targeted and effective intervention strategies.

### 6.3. Individualized Intervention

Another important area for future research is the development of personalized intervention strategies. Each individual has a unique circadian rhythm genotype and microbial composition, which influence their responses to different interventions. By combining microbiomics with circadian rhythm genotypes, it is possible to develop precise intervention strategies based on circadian nutrition and microbial targeting principles [114,115].

### 6.4. Clinical Translation

Clinical translation is a crucial step in applying laboratory research findings to clinical practice. Conducting large-scale randomized controlled trials (RCTs) is essential for verifying the efficacy and safety of interventions such as TRF, probiotics, and melatonin in the treatment of anxiety disorders. RCTs are regarded as the gold standard in clinical research because they can provide reliable evidence regarding the effectiveness of interventions by comparing the intervention measures with a control group. Through well-designed RCTs, researchers can determine the optimal dosage, duration, and combination of intervention strategies for different patient groups. This will help establish evidence-based guidelines for the clinical management of anxiety disorders [116].

By integrating chronobiology and microbiome science, a new paradigm for the prevention and treatment of anxiety disorders is expected to emerge [114,117]. This integrated approach has the potential to provide more effective, personalized, and comprehensive treatment options for individuals with anxiety disorders, thereby improving their quality of life and mental health status.

### 6.5. Conclusions

The circadian rhythm and GM form a dynamic and complex interactive network through neuroendocrine, immune, and neurotransmitter pathways. However, this relationship has not been fully established, and much of the current evidence remains observational or preclinical. Future longitudinal studies and well-designed clinical trials are needed to clarify causality and evaluate intervention strategies. A better understanding of the relationship between the circadian rhythm, GM, and anxiety opens new avenues for the development of intervention strategies.

## Figures and Tables

**Table 1:** Representative gut microbiota findings associated with anxiety-related gut–brain mechanisms.

Study Design	Participants or Model	Anxiety Assessment/ Diagnostic Criteria	Key Microbiota-Related Findings	Ref.
Human cross-sectional study	GAD patients (*n* = 40); healthy controls (*n* = 36)	Clinical diagnosis of GAD	↓ Microbial richness/diversity ↓ SCFA-associated factors, including *Lachnospira, Butyricicoccus*, *Faecalibacterium*, *Eubacterium rectale*, and *Sutterella* ↑ Anxiety-associated dysbiosis profile, including *Fusobacterium*/*Escherichia-Shigella* related factors	[38]
Human clinical microbiome study	Active GAD patients (*n* = 36); healthy controls (*n* = 24); remission assessed after treatment	Clinical diagnosis of active GAD Anxiety severity assessed using Hamilton Anxiety Rating Scale (HAM-A) and Self-rating Anxiety Scale (SAS)	↓ OTU number and α-diversity in active GAD ↓ Firmicutes and *Tenericutes* in active GAD Negative association with anxiety severity: *Eubacterium*, *coprostanoligenes* group, Ruminococcaceae UCG-014, *Prevotella* 9 Positive association with anxiety severity: *Bacteroides*, *Escherichia-Shigella*	[39]
Human shotgun metagenomic study	SAD patients (*n* = 31); matched healthy controls (*n* = 18)	SAD diagnosis confirmed by MINI based on DSM-5 criteria Social anxiety symptoms assessed using LSAS-SR	β-diversity differed between SAD and controls ↑ *Anaeromassilibacillus* and *Gordonibacter* in SAD ↑ *Anaeromassilibacillus* sp. An250 in SAD ↑ *Parasutterella excrementihominis* in controls ↑ Aspartate degradation I module in SAD	[40]
Human population-based 16S rRNA study with null findings	South African adults; self-reported state and trait anxiety symptoms	STAI state and trait anxiety scores, symptom-based, not clinical diagnosis	No significant associations between state or trait anxiety severity and alpha or beta diversity Anxiety-related taxonomic associations not significant after multiple testing correction.	[41]
Human-to-mouse microbiota transfer study	Antibiotic-treated mice receiving SAD- or control-donor microbiota	Human SAD donor diagnosis Social fear sensitivity assessed in recipient mice	SAD-associated microbiota transfer → ↑ Social fear sensitivity Altered central/peripheral immune readouts Altered oxytocin-related signaling	[42]
Germ-free mouse study	Germ-free mice vs. specific-pathogen-free mice	Anxiety-like behavior assessed in germ-free mice	Absence of conventional gut microbiota Altered anxiety-like behavior Central neurochemical changes	[43]
Antibiotic-induced microbiota alteration study	Mice with antimicrobial-induced gut microbiota alteration	Step-down and light/dark preference tests in mice after antimicrobial-induced microbiota alteration	Altered gut microbiota composition Changed exploratory behavior Altered central BDNF-related signaling	[44]
Foundational circadian microbiota study	Mice and humans under feeding-rhythm-related conditions	Anxiety-related outcomes were not assessed Foundational circadian GM rhythmicity study	Feeding rhythm-related diurnal oscillations in gut microbiota composition and function in mice and humans Disruption by host clock disturbance or jet lag, leading to dysbiosis	[45]
Circadian immune mechanism study	Mouse intestinal microbiota–host circadian immunity model	Anxiety-related outcomes were not assessed Diurnal intestinal innate immune rhythms were assessed in mice	Microbiota-dependent diurnal innate immune rhythm Rhythmic epithelial attachment of segmented filamentous bacteria Rhythmic antimicrobial response	[24]
Circadian stress-responsivity study	Microbiota-depleted/microbiota-dependent stress-response mouse models	Stress responsivity assessed in microbiota-depleted or microbiota-dependent mouse models. No clinical anxiety diagnosis	Microbial depletion → disturbed HPA-axis rhythmicity Altered time-dependent stress responsivity Microbiota-dependent circadian regulation of stress response	[28]
GAD microbiota-transfer study	Mice receiving fecal microbiota from GAD-related donors/GAD-associated microbiota model	Human GAD donor microbiota Anxiety-like behavior assessed in recipient mice	FMT-GAD → anxiety-like behavior Altered endocannabinoid-related signaling Changes involving CB1R, FAAH, and MAGL-related pathways	[46]

↑ indicates increased abundance, enrichment, positive association, or enhanced behavioral/functional readout; ↓ indicates decreased abundance, depletion, or negative association. For animal and mechanistic studies, this column reports anxiety-related behavioral endpoints or indicates when anxiety outcomes were not assessed. Not all human studies have reported clear GM differences in anxiety-related conditions. A population-based study assessing state and trait anxiety symptoms found no significant associations between anxiety symptom severity and alpha or beta diversity, and the anxiety-related taxonomic associations did not remain significant after multiple testing correction [41]. These inconsistent findings may reflect differences in sample size, population background, anxiety assessment, sequencing methods, medication exposure, and other uncontrolled factors.

## Data Availability

No new data were created or analyzed in this study. Data sharing is not applicable to this article.
